# Mechanisms and clinical implications in renal carcinoma resistance: narrative review of immune checkpoint inhibitors

**DOI:** 10.20517/cdr.2023.02

**Published:** 2023-06-27

**Authors:** Sunil Samnani, Faraz Sachedina, Mehul Gupta, Edward Guo, Vishal Navani

**Affiliations:** ^1^Department of Internal Medicine, The University of Calgary, Calgary T2N 1N4, Canada.; ^2^Cumming School of Medicine, University of Calgary, Calgary T2N 4N1, Canada.; ^3^Department of Medical Oncology, Tom Baker Cancer Centre, Calgary T2N 4N2, Canada.

**Keywords:** Renal cell carcinoma, immunotherapy, treatment resistance, tumor microenvironment, intrinsic factors

## Abstract

Clear cell renal cell carcinoma (ccRCC) is the most common histological subtype of renal cell carcinoma. The prognosis for patients with ccRCC has improved over recent years with the use of combination therapies with an anti-programmed death-1 (PD-1) backbone. This has enhanced the quality of life and life expectancy of patients with this disease. Unfortunately, not all patients benefit; eventually, most patients will develop resistance to therapy and progress. Recent molecular, biochemical, and immunological research has extensively researched anti-angiogenic and immune-based treatment resistance mechanisms. This analysis offers an overview of the principles underpinning the resistance pathways related to immune checkpoint inhibitors (ICIs). Additionally, novel approaches to overcome resistance that may be considered for the trial context are discussed.

## INTRODUCTION

Renal cell carcinoma (RCC) accounts for 3%-5% of all malignancies in adults, with an incidence of about 400,000 cases yearly and mortality in approximately 175,000 cases worldwide in 2020^[1-4]^. Around one-fourth of patients present initially with metastasis, with the rest recurring after nephrectomy^[5]^.

The most common histological pattern of renal tumors is clear cell renal cell carcinoma (ccRCC), which accounts for 70%-90% of the disease, followed by papillary (10%-15%) and chromophobe RCCs (3%-5%)^[6,7]^.

In the past few decades, the mainstay of treatment of metastatic RCC (mRCC) involved vascular endothelial growth factor receptor tyrosine kinase inhibitors (VEGFR-TKI)^[8,9]^. Monoclonal approaches to the same target, as characterized by bevacizumab, have been unsuccessful. A study by Motzer *et al.* showed no improvement in OS in patients with mRCC treated with atezolizumab plus bevacizumab^[10]^. When this immunologically “hot” tumor microenvironment is exposed to immune checkpoint inhibitors and/or inhibitors of VEGF as a combination, outcomes are significantly and clinically improved compared to VEGFR-TKI monotherapy alone^[7,11]^. Unfortunately, a cure is still not achievable for the vast majority of these patients presenting in the metastatic setting.

This review aims to summarize the molecular mechanisms that drive resistance to immune checkpoint inhibitors and their clinical implications in patients with mRCC.

### Molecular mechanism of resistance to immune checkpoint inhibitors

Resistance to ICIs therapy has been classified into primary (initial) resistance and secondary (acquired) resistance^[12]^. Part of the classification is based on imaging response to ICI therapy, defined by the Response Evaluation Criteria in Solid Tumors (RECIST) v1.1 criteria^[13]^. Primary resistance is defined as the best response of progressive disease (PD) or stable disease (SD) for less than 6 months of ICI initiation. Patients with secondary resistance develop progressive disease after having a response or stable disease for greater than six months. Various mechanisms have been postulated for developing primary and secondary resistance in solid tumors, including mRCC, which can be broadly subcategorized into patient-related factors, tumor cell-related factors, or factors related to the tumor microenvironment (TME). The precise role of these mechanisms and a variety of others in primary and secondary resistance in the context of mRCC remains to be fully elucidated. However, a better understanding of these complex mechanisms is crucial to optimizing treatment approaches for those resistant to mRCC disease^[14]^.

### Patients’-intrinsic factors

Patients’-intrinsic factors play a vital role in the immune response to the therapy. Several factors are related to the immune response in cancer therapy and are summarized in Table 1.

**Table 1:** Molecular mechanism of resistance - patient’s intrinsic factors

**Factor**	**Theorized mechanism**	**Potential outcomes**
Gender^[15-18]^	Exposure to mutagenic factors (i.e., Smoking, exposure is higher in men)	Differences in tumor histology
	Estrogen modulation leads to: (a) Increased expression of FoxP3(b) Modulation of PD-1 expression	Increased regulatory T-cell, dendritic cell, and macrophage activity.
Obesity^[19]^	Both pro-tumor and anti-tumor activity	Mixed outcomes in patients with RCC
Sarcopenia^[95]^	Low skeletal mass index	Worse prognosticator
HLA genotype^[25,26]^	Classical HLA-1 Molecules resulting in High Divergent Expression	Greater response to ICI treatment
Gut microbiome^[27,28]^	Fecal Microbiota Transplant resulting in upregulation of CD4+ T cell and PD-L1	Improved ICI response
Antibiotic/Steroid use^[29,33]^	Antibiotic Use causing dysbiosis	Decreased ICI response
	Steroids altering T-cell activation, altering gut microbiota	May result in favorable disease course due to other factors

PD-1: Programmed cell death protein 1; HLA: human leucocyte antigen; ICI: immune checkpoint inhibitors.

#### Gender

Gender-related factors may be attributed to confounding behaviors, such as male patients with increased exposure to smoking^[15]^. Another possible hypothesis is the role of estrogen modulation. Estrogen upregulates the activities of T-cells and PD-1 expression on cells^[16,17]^. A recent meta-analysis showed a significant disparity in overall survival (OS) between men and women with metastatic cancers with pooled OS in men was HR = 0.72 (95%CI: 0.65-0.79) and in women was HR = 0.86 (95%CI: 0.79-0.93), with a positive interaction value^[15]^.

In a randomized trial in patients with ccRCC, treated with nivolumab or everolimus in a second-line setting, the hazard ratio (HR) for death in males was 0.70 (95%CI 0.50-0.90) compared to 0.84 (0.57-1.24) in women, which was similar to patients with melanoma or small cell lung carcinoma treated with ICIs^[18]^.

#### Obesity

Obesity has shown variable modification capabilities in a human cell with both pro-tumor (increased VEGF, vascular endothelial growth factor, hypoxia, angiogenesis, plasmacytoid dendritic cells, and mast cell infiltration) and anti-tumor activities (increased T-cell and natural-killer cell response and decrease intratumor PDL-1 expression)^[19]^. Several studies have shown the beneficial effects of obesity on survival in patients with RCC^[20,21]^. Utilizing CT based body composition, correlations with specific phenotypes were found (i.e., Adipose, skeletal density vs. sarcopenia). While the exact mechanism to an increased BMI results in positive outcomes, theories include tumorigenic immune dysfunction *vs.* T-cell antigen cross-reactivity. This has the potential to improve sensitivity to ICI therapy. In contrast, an analysis of those with a similar phenotypic profile who were receiving Anti-PD-1 therapy showed a reduction in therapeutic response (lower PFS) as compared to their leaner counterparts^[22-24]^.

#### Malnutrition and sarcopenia

Sarcopenia has been associated with adverse outcomes even before the immunotherapy era^[22-24]^. A retrospective multicenter real-world study found that sarcopenic patients had significantly worse OS (HR = 2.2, 95%CI: 1.3-3.6, *P* = 0.0026) when treated with ICIs. On multivariate analysis, low muscle mass was associated with an inferior OS^[25]^. Similarly, malnutrition has shown poor prognosis and quality of life in patients with lung cancer and is useful in predicting the response to the treatment^[26,27]^.

#### Human leucocyte antigen genotype

Human leucocyte antigen (HLA) class 1 genes usually underlie the control of cancer with the diverged presentation of proteins that can influence response to ICIs^[25,26]^. This hypothesis suggests that patients with heterozygous divergent alleles have a more extensive presentation of peptides to assist T-cell response than less divergent HLA alleles. Patients with melanoma having high divergent alleles receiving ICIs respond better to the treatment. The outcomes were significantly different with 20 months in patients with high divergent alleles than 8 months in patients with low-divergent alleles (HR = 0.43, 95%CI: 0.2-0.8, *P* = 0.0094)^[26]^. Similar outcomes were observed in non-small cell lung cancer patients who received ICIs^[25,26]^. So far, there is a lack of specific studies for patients with RCC to understand the difference between high versus low divergent alleles and their response to the treatment^[25,26]^.

#### Gut microbiota

Fecal microbiota transplantation (FMT) has been studied as a predictive factor for a response to ICIs treatment. Routy *et al*. found a positive correlation between response to ICI and Akkermansia muciniphila in mice receiving anti-PD-1 blockade^[27]^. It has also been noticed that the FMT from a patient who responded to ICIs in germ-free mice improves ICI efficiency but, unfortunately, not in non-responding patients. FMT upregulates helper T-cells (CD4+) and natural killer T- cells (NKT) in the spleen, resulting in improved response to ICIs. In a prospective randomized control trial, the gut microbiome modulation in patients with mRCC has been associated with improved PFS^[28]^, which will be discussed later in this paper.

#### Antibiotics and use of steroids

The alteration in the gut microbiome predicts the response to immunotherapy. It has been observed that dysbiosis with antibiotics has been associated with poor outcomes in patients treated with ICIs^[29]^. Other retrospective studies by Lalani^[30]^, Derosa *et al.*^[31]^, and Tinsley *et al.*^[32]^ also showed a negative association between antibiotic use and outcomes in patients with RCC, with a significant decrease in PFS^[30-32]^. Derosa *et al.*^[31]^ and Tinsley *et al.*^[32]^ also showed a reduction in OS. On multivariate analysis, antibiotic use was an independent predictive factor for worse outcomes in patients with RCC^[31,32]^.

The use of steroids is routine in oncology patients for symptom management. A dose of > 10 mg prednisone or equivalent induces immunosuppression by altering the T cell activation with the expansion of M2 macrophages and thus altering the gut microenvironment. In a multicentre study by Arbour *et al.*^[33]^, baseline higher steroid dose was associated with lower ORR (7% *vs* 18%), progression-free survival (PFS) (*P* < 0.001), and OS (*P* < 0.001). However, steroids used to manage immune-related adverse events have shown no negative impact on the efficiency of ICIs^[34-36]^. Indeed, patients that develop severe immune-related adverse events and need systemic steroids have a more favorable disease course in recent real-world data^[37]^.

## TUMOR CELL INTRINSIC FACTORS

Tumor cell-intrinsic factors are cell-related factors that can identify the response to the therapy and are summarized in Table 2.

**Table 2:** Molecular mechanism of resistance - tumor cell-intrinsic factor

**Factor**	**Theorized mechanism**	**Potential outcomes**
IFN-γ dysregulation^[39-41]^	Loss of JAK-1&2 Function resulting in decreased PD-L1 expression	Resistance to PD1 Inhibitors
	Decreased immune cell recruitment with decreased MHC-1 antigen presentation	Increased proliferation and decreased apoptosis
MAPK^[38,42]^	Increased production of VEGF, IL-6,8 and 10 inhibiting T-cell function	Increased resistance
	Downregulation of antigen/MHC expression	Increased proliferation and decreased apoptosis
PI3K/AKT alteration^[43,44]^	Loss of expression of PTEN resulting in:Cytokine suppressionInhibition of auto-phagosome activity	Decrease ICI response due to poor recruitment.
Wnt/β-catenin overexpression^[45,46,48]^	Absence of T-cell expression and T-cell exclusion	Resistance to ICI therapy
Loss of MHC^[96-99]^	Loss of MHC-1 and 2	Resistance to ICI therapy

IFN-γ: Interferon-gamma; PD-L1: programmed death ligand 1; JAK: janus kinase; MHC: major histocompatibility complex; VEGF: vascular endothelial growth factor; IL: interleukin; PI3K: phosphoinositide 3-kinases; PTEN: phosphatase tensin homolog; ICI: immune checkpoint inhibitors; MAPK: mitogen-activated protein kinase.

### Signalling pathways

#### Interferon-gamma signaling pathway

The activation of interferon-gamma (IFN-γ) activates Janus kinase (JAK), and interferon regulatory factor 1 (IRF1), leading to the expression of PDL1. Patients with dysregulation of the IFN-γ pathway were shown to develop resistance to ICIs^[38]^. The IFN-γ recruits immune cells and enhances MHC-1 antigen presentation, which is essential for the antiproliferative and proapoptotic signals^[39,40]^. The dysregulation of JAK genes has been associated with a lack of response to the IFN-γ and, thus, with PD1 inhibitors^[41]^.

#### Mitogen-activated protein kinases

Mitogen-activated protein kinases (MAPK) is associated with VEGF, IL-6, IL-8, and IL-10 production, inhibiting T-cell functions and immune cell recruitment. The MAPK pathways downregulate antigen presentation and MHC expression and decrease the sensitivity to antiproliferative effects of IFN-γ and TNF alpha^[38,42]^.

#### PI3K/mTOR/AKT pathway

Patients with ccRCC have altered PI3K/AKT pathway along with alteration in the expression of PTEN^[43]^. These lead to the inhibition of immunosuppressive cytokines and auto phagosome, resulting in decreased T cell response, cell recruitment, and cell-mediated death. The loss of PTEN has been associated with the worse outcome in patients treated with ICIs therapy^[44]^.

#### Wnt/β-catenin pathway

Wnt/β -catenin is involved with stem cell embryogenesis, immune regulation, and cell differentiation. In most cancers, it is overexpressed, reducing the expression of T cells and thus resulting in resistance to ICIs^[45-48]^.

### Insufficient tumor antigenicity

Investigations highlighted a correlation between poor antigenicity and decreased sensitivity to ICIs^[49,50]^. Repeat analysis supports the idea that these tumor neoantigens can be targeted to promote a positive response to checkpoint blockade. This concept can be applied to a variety of malignancies.

It is important to highlight that despite an understanding of the molecular patterns of resistance to ICIs in this context, no current biomarkers, molecular or otherwise, are validated for use in the clinic to help determine therapy selection.

## TUMOR MICROENVIRONMENT

Tumor microenvironment (Tme) includes tumor cells and other cells interacting with them. These cells are crucial for tumor cells’ development, progression, and relapse. Factors that can affect the TME are summarized in Table 3.

**Table 3:** Molecular mechanism of resistance - tumor microenvironment

**Factor**	**Theorized mechanism**	**Potential outcomes**
Tumor-associated Macrophage activation^[51-53]^	Greater M1 and M2 Macrophage activation, M2 (CD163+) subset correlated to poor survival outcomes	Nivolumab improved PFS in high M2 density activation
High T-cell density^[56]^	Higher CD8+ infiltration associated with poorer clinical outcomes in ccRCC patients	Prognosticator for worse potential outcomes
B-cell and tertiary lymphoid structures^[50,57,62]^	Increased B cell-related genes associated with strong memory response	Great response to therapy
	Tertiary lymphoid structures promote Regulatory T-cell activation	Prognosticator for positive potential outcomes.
Hypoxia^[64-67]^	Increased recruitment and infiltration of myeloid-driven suppressor cells, decreased function of cytotoxic T-cells	Immune evasion
	Induction of HIF-1a and 2a, increasing PD-L1 expression, and VEGF generation	Immune evasion

PFS: Progression-free survival; ccRCC: clear cell renal cell carcinoma.

### Tumor-associated macrophage

During inflammation, macrophages transform to M1 (classical) or M2 (alternative) activation. M1 produces inflammatory cytokines (ie. IL-6, IL-12, and IL-23), whereas the later correlates with the subsets of M2a, M2b, M2c, and M2d, respectively^[51,52]^. In 2011, a study found poor survival in patients with RCC with a high burden of M2 (CD163+)^[53]^. The diverse and extensive tumor-associated macrophages in the TME of patients with RCC indicate cancer progression and metastases. Strategies to direct therapeutic response to suppress tumor-associated macrophage recruitment have shown some improvement in the response^[54]^, but macrophage-targeted therapies still need to be implemented in clinical settings

.

The TME associated with abundant cells has shown an overall better response to ICIs (*P* < 0.001) as compared to TKI treatment (*P* = 0.15)^[55]^. Voss *et al*. found that the TME, with abundant infiltrative cell types, was associated with a good response to ICIs (*P* < 0.001), which was the opposite in patients treated with TKIs (*P* = 0.15)^[55]^. In another trial, patients treated with nivolumab having higher densities of M2 macrophages showed better PFS (HR = 0.69, *P* = 0.016), but no significant improvement in OS (*P* = 0.5)^[56]^.

### T cells

RCC is a tumor mainly enriched with T cells, particularly CD8+ T cells. The presence of CD8+ T cells is correlated with poor outcomes^[57,58]^. Choueiri *et al.* reported that high CD8 infiltration was associated with poor clinical outcomes in patients with ccRCC treated with sunitinib versus the avelumab-axitinib combination^[59]^. This was discordant from another phase II study, NIVOREN-GETUG AFU 26, which showed that patients with high CD8+ infiltration treated with Nivolumab have poor PFS (HR = 3.96, *P* < 0.0001) and OS (HR = 2.43, *P* = 0.04)^[56]^. Interestingly, Voss. *et al.* identified no correlation between the CD8+ cell infiltrations and survival in mRCC when treated with ICIs^[55]^.

The Cancer Genome Atlas analysis showed that patients with RCC have more regulatory T-cells and are associated with poor clinical outcomes (HR = 1.59, 95%CI: 1.23-0.06; P < 0.01)^[60]^.

### B cells and tertiary lymphoid structures

B cells assist T-cell regulation and differentiation through different pathway activation, including interleukin (IL) and growth factors^[49]^. The memory of classical B cells has been associated with response against tumor-associated antigens^[50]^. The Microenvironment Cells Populations-counter (MCP-counter) analysis showed that responders have higher B cell densities in the TME than non-responders in melanoma and ccRCC^[61]^.

Tertiary lymphoid structures are also associated with better outcomes in oncology patients. It assists in transforming cells and activating T regulatory cells^[57,62]^.

### Hypoxia

Tumors in the hypervascular environment led to insufficient nutrition and oxygen intake, resulting in hypoxia and progression^[63]^. Hypoxia induces upregulations of myeloid-driven suppressor cells that lead to decreased function of cytotoxic T cells^[64,65]^. Hypoxia also induces factor 1a (HIF-1a) and 2a (HIF-2a) to express PD-L1 in tumor cells^[66,67]^. Elevated levels of HIF are associated with generating VEGF, which acts as an immune escape mechanism

by upregulating CTLA4, TIM3, LAG3, and PD-L1 on dendritic cells^[65,68]^. Hypoxia causes tissue deposits of adenosine which suppresses the activity of T cells^[69]^.

## CLINICAL IMPLICATION OF ICIs RESISTANCE

### ICI resistance in the clinical trial setting

Despite therapeutic advances, ICI resistance remains a significant issue in clinical trials and real-world settings due to a lack of validated clinical tools to identify patients at risk for primary or secondary resistance. Currently, there is no clinically accessible molecular classification of mRCC, and although clinical trials investigating molecular biomarkers in mRCC populations are promising, such approaches remain restricted to the research domain.

### Molecular subtypes as biomarkers of ICI response

Using molecular markers to identify subgroups of mRCC patients who experience more robust responses to ICI therapy remains a promising avenue of study.

Transcriptomic analysis in a subset of primary resected ccRCC samples obtained from patients with metastatic disease identified a 35-gene signature capable of subgrouping metastatic clear-cell renal cell carcinoma (ccmRCC) into four groups (ccRCC 1-4). These subtypes were associated with differential response to sunitinib treatment, with ccRCC 1 and 4 tumors having a lower response rate and poorer survival outcomes than ccRCC 2 and ccRCC 3 subtypes. Molecular characterization of these tumor subtypes identified unique tumor microenvironments that may explain the relative response to sunitinib treatment. For example, ccRCC 4 cancers were associated with a suppressive immune microenvironment with overexpression of PD-L1 and PD-L2, while the ccRCC 2 subgroups demonstrated a more pro-angiogenic subtype^[70]^. These results lead to the hypothesis that these molecular subtypes could stratify patients into groups most likely to benefit from TKI therapy, ICI monotherapy, and combined ICI therapy approaches.

This hypothesis was tested in the phase II BIONIKK trial, the first to stratify patients into treatment groups based on their molecular subtype. Patients were divided into the ccRCC risk group using the aforementioned 35-gene transcriptomic signature and were prospectively allocated into treatment groups. Patients with ccRCC 1 or 4 tumors were assigned to anti-PD1 monotherapy with nivolumab alone or anti-PD1/anti-CTLA4 with a combination of nivolumab-ipilimumab. Those patients with ccRCC 2 or ccRCC 3 group tumors received TKI therapy and a combination of nivolumab-ipilimumab. The primary outcome of this study was the overall response rate per the RECIST criteria, with survival outcomes, tolerability, and duration of the response being secondary outcomes^[71]^. Meylan *et al.*^[72]^ further identified the biomarkers for the efficacy of Nivolumab (N) +/- Ipilimumab (I) in mRCC patients with TLS > 2 treated with N or NI showed a response rate of 73% and 71%, respectively. Both TLS > 2 and higher densities of Ki67/PD1 correlated with better response rates (80% *vs.* 43% *P* < 0.01) and decreased incidence of progression events (5% *vs* 36%, *P* = 0.02).

Another example of molecular subtyping in a trial setting comes from the IMmotion 150 and 151 study and the ongoing phase II OPTIC trial. The IMmotion 150 trial compared treatment with atezolizumab alone or in combination with bevacizumab to sunitinib in a cohort of 305 patients with previously untreated metastatic renal cell carcinoma. Though treatment with a combination of atezolizumab alone or in combination with bevacizumab did not demonstrate improved PFS, the retrospective analysis did identify gene expression signatures associated with PFS involving angiogenesis, immunity (primarily T-effector presence and function), and myeloid inflammatory pathways. In particular, patients with a high angiogenesis signature score had an improved overall response rate and a longer PFS within the treatment sunitinib arm compared to those with low signature scores. Those with a high T-effector gene signature score experienced an improved overall response rate and longer PFS than those with a low score in the atezolizumab plus bevacizumab arm. In contrast, those with a high myeloid inflammation gene expression score experienced a shorter PFS in the atezolizumab plus bevacizumab and atezolizumab monotherapy treatment arms. These results suggest that such gene expression signatures may be useful tools in identifying patients more likely to benefit from various treatment regimens^[73]^.

The findings of the IMmotion 150 study were validated and expanded upon in the phase 3 open-label IMmotion 151 study, which enrolled 915 patients and allocated them to receive either a combination of atezolizumab plus bevacizumab or sunitinib monotherapy. While this trial failed to demonstrate an overall survival benefit to combination therapy, it has provided rich biomarker data for mRCC molecular subtyping^[74]^. The IMmotion 151 trial retrospectively assigned patients to subgroups based on the previously identified gene expression signatures developed in the IMmotion 150 cohort. The authors again demonstrated that patients with a high angiogenesis signature score had improved PFS in the sunitinib arm, and those treated with atezolizumab plus bevacizumab had an improved PFS compared to sunitinib in subgroups with a high T-effector score or a low angiogenesis score. Based on these analyses, patients enrolled in the IMmotion 151 trial were further retrospectively classified into seven molecular subtypes - termed clusters - according to their unique genomic and transcriptomic enrichment pathways. Clusters 1 (angiogenic/stromal) and 2 (angiogenic) were enriched among the favorable risk groups as defined by the IMDC and Memorial Sloan-Kettering Cancer Center (MSKCC) scores, while clusters 4 (T-effector/proliferative), 5 (proliferative), and 6 (stromal/proliferative) were enriched among poor-risk patients.

Additionally, patients in clusters 1 and 2 demonstrated improved survival, while those in cluster 6 had poor PFS outcomes compared to other clusters, irrespective of treatment arm. Moreover, treatment with atezolizumab plus bevacizumab demonstrated improved overall response rates and longer PFS than sunitinib in clusters 4, 5, and 7 (small nucleolar RNA). Multivariable analysis of these identified molecular subtypes with clinical scores, including the MSKCC and IMDC scores, demonstrated an independent association with survival. These results suggested that these molecular subtypes may have the predictive capacity and could be incorporated with existing clinical tools to provide additional benefit, though additional prospective testing would be important prior to any clinical translation^[75]^. Of note, the phase II OPTIC trial is taking the first steps towards this goal, looking to utilize these molecular subgroups to prospectively assign patients to combination ICI treatment with ipilimumab plus nivolumab or ICI-TKI therapy with nivolumab and cabozantinib. Currently, in the recruitment phase, this trial will provide critical insights into the utility of molecular subtypes for treatment stratification in mRCC (NCT05361720).

### Future perspectives

The above trials are a testament to the advancements in understanding the molecular underpinnings of mRCC and the role these insights can play in tailoring therapy in this patient population. Despite this progress, there are still several areas requiring further study. First, many unanswered questions remain regarding integrating these molecular subtypes with current clinical risk criteria. Further, these approaches are time-consuming and cost-prohibitive due to their reliance on multi-omic profiling. Therefore, studies on the cost-efficacy and accessibility of these tools will be critical before a transition into the clinical landscape. Additionally, molecular signature identification may benefit from targeted therapy, as there are an increasing number of TKI and PD-1/PD-L1 options available. Therefore, although these trials’ results are promising, further study is imperative to bring molecular subtypes into the clinical setting.

### Clinical implication in real-world settings

#### Combination of lenvatinib plus everolimus for those with primary resistance

In a cohort of 7 patients that had shown resistance to VEGF-targeted TKI’s or ICI therapy, a combination of lenvatinib and everolimus as either second or third-line therapy resulted in a partial response in three patients and stable disease in three patients. Progression-free survival ranged from 3 to 15 months^[76]^.

#### Combination therapy as second-line therapy active in distinct clinicopathological features

This combination retrospective study included 343 patients, with 123 receiving Cabozantinib and 220 receiving Nivolumab. Patients receiving Nivolumab and first treated with Pazopanib showed a non-statistically significant median overall survival of 26.8 *vs.* 11.6 months. The OS for patients with Cabozantinib was 25.7 months as compared to Sunitinib 21.7 months, but again, not statistically significant (*P* = 0.45). Notably, Cabozantinib exhibited activity in terms of progression-free survival, particularly in patients with Clear Cell histology (7.8 *vs.* 5.4, *P* = 0.026) and those with good risk features (12.3 *vs*. 5.7, *P* = 0.022)^[77]^.

Another phase II trial, including a high dose of Cabozantinib with atezolizumab therapy (COSMIC 021), demonstrated an encouraging clinical response^[78]^. However, the CONTACT-03 study did not show any promising results in patients treated after disease progression during or after immune checkpoint inhibitor therapy (either combination or monotherapy) (NCT04338269).

## APPROACH TO OVERCOME ICI RESISTANCE

### Targeting the TME

#### Colony stimulating factor 1 receptor inhibitor

M2 macrophages promote tumor neoangiogenesis, and progression, which play a role in the treatment’s resistance. The expression of colony stimulating factor 1 receptor (CSF1R) allows switching the type 1 macrophages to the type II tumor associated-macrophages^[79]^. There are phase-1 trials currently undergoing to assess the effectiveness of combining treatment with CSF1R inhibitors and ICIs (NCT02718911, NCT02526017).

#### Indoleamine 2,3-dioxygenase 1 inhibitors

The indoleamine 2,3-dioxygenase 1 inhibitors deprives T cells of nutrients and can be the target for treatment. A study published in 2018 on patients with metastatic ccRCC treated with Nivolumab has shown that IDO-1 overexpression ( > 10%) was found in patients with an excellent response to the treatment and thus better PFS. This study suggested that IDO could be used as a biomarker for patients with RCC^[80]^.

A phase I/II ECHO-202/KEYNOTE 037 trial combining oral IDO-1 enzyme inhibitors with pembrolizumab was associated with a 40% objective response (8 complete and 13 with stable disease)^[81]^. Unfortunately, in another phase III study, IDO-1 enzyme inhibitors failed to show a positive response in patients with melanoma, so their use was stopped after the study results^[82]^.

#### Stimulators of interferon genes and retinoic acid-inducible gene 1 agonists

The stimulators of interferon genes (STING)pathway promotes the production of pro-inflammatory cytokines^[83]^. RIG-1 stimulates natural killer cells and CD8+ T cells^[84]^. There are a c ouple of trials assessing STING agonist and retinoic acid-inducible gene (RIG-1) agonist use as monotherapy or in combination with ICIs (NCT03010176 and NCT 03739138)

#### Targeting HIF-2α

Belzutifan is a first-in-class novel therapy to inhibit HIF-2α resulting in anti-tumor activity by impairing the hypoxic signal pathway in cancer cells. A phase 1 study with patients with pre-treated ccRCC showed a 25% response rate^[85]^. In a phase 2 study, patients who received prior treatments (immunotherapy or chemotherapy) were treated with Belzutifan plus Cabozantinib showed an objective response rate in 16 [30.8% (95%CI: 18.7-45.1)] of 52 patients^[86]^.

Another inhibitor of HIF is PT2385, which was studied in combination with Nivolumab in patients with mRCC who previously had received up to three treatments. It showed an objective response rate of 22% with a PFS of 10 months among patients receiving therapeutic doses of PT2385 versus 4.7 months in the sub-therapeutic group^[87]^.

#### Pegylated IL-2 and cytokines

IL-2 has shown anti-tumor potential by lysing tumor cells^[88,89]^. In a study of patients with mRCC, high-dose IL-2 combined with Pembrolizumab has shown an objective response rate (ORR) of 69%^[90]^. In a study in patients with previously untreated mRCC, Bempegaldesleukin (NKTR-214), combined with Nivolumab, showed an ORR of 54% in untreated mRCC^[91]^.

### Targeting patient’s intrinsic factor

#### Modulation of the gut microbiome

CBM58 is a bifidogenic live bacterium that can augment the effect of ICI by modulating the gut microbiome. The single-center randomized study (NCT03829111) in mRCC patients assessed nivolumab and ipilimumab with or without daily oral CBM588. The abundance of the bifidogenic bacterium was not seen. However, patients who received nivolumab-ipilimumab with CBM 588 had a significantly lower PFS (12.7 months *vs.* 2.5 months, HR 0.15, 95%CI: 0.05-0.47, *P* = 0.001) and a higher response rate (58% *vs.* 20%, *P* = 0.06) compared to those without CBM 588^[92]^.

Recently Fecal microbiota transplantation (FMT) has been shown in several studies to augment the effect of ICI and overcome resistance, particularly in patients with melanoma^[27,93,94]^. Clinical trial in patients with RCC is still recruiting to assess the role of FMT in improving the efficacy of ICI (NCT04758507).

## CONCLUSION

The outcomes of patients with metastatic RCC have changed significantly over the past few decades. The new therapeutic options, particularly with ICIs, have survival benefits and a durable response rate either used as a monotherapy or in combination with other therapies. However, patients may have resistance initially reflecting primary resistance or initial response to the treatment and then develop secondary resistance. Resistance to ICIs is influenced by three significant components: patient intrinsic factor, tumor cell-intrinsic factor, and contributions from the tumor microenvironment. Many innovative approaches have been studied and investigated in clinical trials to assess ICI resistance mechanisms in patients with mRCC.

## DECLARATIONS

### Copyright

© The Author(s) 2023.
